# A survey to explore the psychological impact of the COVID‐19 pandemic on radiation therapists in Norway and Canada: A tale of two countries

**DOI:** 10.1002/jmrs.557

**Published:** 2021-10-29

**Authors:** Sara Morassaei, Lisa Di Prospero, Elisabeth Ringdalen, Sunniva S. Olsen, Agnethe Korsell, Darby Erler, Carmen Ying, Sang Ho Choi, Amanda Bolderston, Jacqueline Middleton, Safora Johansen

**Affiliations:** ^1^ Practice‐Based Research and Innovation Sunnybrook Health Sciences Centre Toronto Ontario Canada; ^2^ School of Rehabilitation Therapy Queen’s University Kingston Ontario Canada; ^3^ Department of Radiation Oncology, Temerty Faculty of Medicine University of Toronto Toronto Ontario Canada; ^4^ Health Faculty Oslo Metropolitan University Oslo Norway; ^5^ Odette Cancer Centre Sunnybrook Health Sciences Centre Toronto Ontario Canada; ^6^ Radiation Therapy Program, Department of Oncology University of Alberta Edmonton Alberta Canada; ^7^ Cancer Treatment Department Oslo University Hospital Oslo Norway

**Keywords:** Coronavirus, health personnel, radiotherapy, mental health, quality of life, psychological stress, anxiety

## Abstract

**Introduction:**

Several studies have demonstrated the psychological impact of the COVID‐19 pandemic on health care providers. However, there is little known about how the COVID‐19 pandemic has impacted radiation therapists (RTs) in Norway or Canada. The aim of this investigation was to study the psychological impact of working during the COVID‐19 pandemic among RTs in Canada and Norway.

**Methods:**

Online surveys were administered to a convenience sample of RTs and RT department managers. Approximately 2000 and 300 RTs were invited to participate from Canada and Norway, respectively. The RT survey collected information on demographics, work‐related stressors, psychological impact, quality of life, and workplace support programmes. The RT manager survey collected information on departmental changes, patient volumes, staff shortages and redeployment, personal protective equipment, and infection control measures. Descriptive analysis, group comparisons and logistic regression were used to examine the impact of COVID‐19 on RTs in the two countries, while open‐ended questions were examined through thematic analysis.

**Results:**

Work‐related stress and anxiety were prevalent among Canadian (*n* = 155) and Norwegian RTs (*n* = 124), with Canadian RTs reporting higher levels. Fear of transmission, changes in PPE usage, and changes in staffing were reported as the most frequent work‐related stressors. Themes related to working during the pandemic included: generalised anxiety; physical, emotional and cognitive symptoms of stress; and loneliness, as well as negative impact on health and quality of relationships. Survey findings from RT department managers in Canada (*n* = 12) and Norway (*n* = 13) suggest that the pandemic had an organisational impact on RT departments due to implemented infection control measures and changes in staffing.

**Conclusion:**

The COVID‐19 pandemic has led to similar stressors amongst Canadian and Norwegian RTs but relatively higher levels of psychological impact among Canadian RTs. Findings demonstrate the importance of mental health support programmes in the workplace to mitigate the psychological impact of the COVID‐19 pandemic on RTs.

## Graphical abstract

The overall aim of this investigation was to study the psychological impact of working during the COVID‐19 pandemic among RTs in Canada and Norway. Work‐related stress and anxiety were prevalent among Canadian and Norwegian RTs, with Canadian RTs reporting higher levels. Fear of transmission, changes in PPE usage, and changes in staffing were reported as the most frequent work‐related stressors. The findings suggest that the COVID‐19 pandemic has led to similar stressors amongst Canadian and Norwegian RTs but relatively higher levels of psychological impact among Canadian RTs.

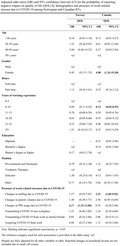

## Introduction

Due to the COVID‐19 pandemic, major changes have been observed throughout hospital environments around the world. Radiation oncology treatment has continued throughout the pandemic; thus, radiation therapists (RTs) have been important frontline healthcare providers (HCPs) responsible for the delivery of daily radiation treatments. Previous research from past epidemics as well as recent research on the current COVID‐19 pandemic indicates that HCPs around the globe can experience changes to their mental health as a result of working during these events.^1^, ^2^, ^3^, ^4^

In 2003, at the time of the SARS outbreak, it was reported that HCPs experienced emotional stress and concern for personal and familial health.^1^ HCPs also reported feelings of stigmatisation and rejection in their community, fear of infection, and reluctance to work.^2^ The SARS epidemic also demonstrated the possibility of long‐term psychological impact on HCPs.^3^ Psychological impact can be characterised as significant experiences of stress, anxiety, depression, and insomnia, among other mental health symptoms.^3^, ^4^, ^5^, ^6^, ^7^ One year after the outbreak, HCPs reported elevated levels of stress, anxiety, and depression.^3^ Furthermore, being quarantined during the outbreak increased the likelihood of post‐outbreak depressive symptoms, and moreover, being exposed to highly stressful work situations puts HCPs at risk of post‐traumatic stress disorder (PTSD).^4^, ^7^ Given that HCPs were psychologically impacted from smaller scale outbreaks indicates that it is extremely likely that larger scale outbreaks such as the COVID‐19 pandemic puts HCPs at risk of developing psychological distress.^4^, ^7^

Several studies have been published demonstrating the psychological impact of the COVID‐19 pandemic on HCPs. A recent systematic review by Pappa et al. found elevated presence of anxiety, depression, and insomnia in HCPs during the COVID‐19 pandemic.^5^ Huang et al. also reported increased symptoms of depression, anxiety, insomnia, and distress among HCPs due to the pandemic.^6^ Factors identified as increasing the risk of experiencing psychological symptoms included being a woman, those in a nursing role and HCPs directly engaged in the care of COVID‐19 patients.^6^, ^8^ The evidence published to date has focused on doctors and nurses with limited literature focused on allied health professionals practicing as radiographers or radiation therapists.^5^, ^7^

A study by Akudjedu et al. assessed the impact of the COVID‐19 pandemic on UK radiography practices.^9^ Radiographers and RTs reported increased work‐related stress with fear of infection and perceived inadequate personal protective equipment (PPE) acting as key contributors to stress.^9^ Similar studies have been carried out focused on the radiology workforce in India and in a selection of North African and Middle Eastern countries,^10^ as well as on Australian radiographers and RTs.^11^ Both studies reported an increase in the level of anxiety and work‐related stress among respondents that were directly impacted by the COVID‐19 pandemic.^10^, ^11^

To our knowledge, there is little known about how the COVID‐19 pandemic has impacted Norwegian RTs and Canadian RTs.^12^ Previous surveys by the Canadian Association of Medical Radiation Technologists (CAMRT) examined the effects of the pandemic on its members, including understanding any policy, workload, and patient care changes from the perspective of management.^12^ However, it is difficult to assess the psychological impact on Canadian RTs based on the data captured. Similarly, there is little known about how the COVID‐19 pandemic has impacted Norwegian RTs. These two high‐income countries are comparable in their universal‐access healthcare systems, medical infrastructure, and available resources.^13^ While international travel restrictions were imposed in both countries during the COVID‐19 pandemic, Norway imposed a national lockdown to limit the spread of COVID‐19, while lockdown restrictions were managed at a provincial level in Canada. Norway has one of the lowest COVID‐19 death rates (0.59% as of July 2021) among its social democratic counterparts in the northern European region and compared to Canada (1.9%).^14^ While Canada fared much better than other liberal democracies such as the United States and United Kingdom in terms of number of cases, there have been approximately 3995 cases versus 2545 cases per 100,000 population in Canada and Norway, respectively.^14^ Reporting on these two countries provides an opportunity to understand the relative impact of the pandemic on RTs using two international counterparts. Therefore, the overall aim of this investigation was to study the psychological impact of working during the COVID‐19 pandemic among RTs in Canada and Norway.

## Methods

The psychological impact of the COVID‐19 pandemic on Canadian and Norwegian RTs was investigated by surveying RTs and RT department managers.

### Study population

All practicing RTs and RT department managers in Canada (estimated to be approximately 2000 potential respondents) and Norway (estimated to be approximately 300 potential respondents) were invited to participate in the study. Inclusion criteria included those who were working since before October 1, 2019, as to adequately assess the psychological impact of the emerging COVID‐19 pandemic. An email was sent to RT department managers in early January 2021 explaining the purpose of the study with instructions to distribute the electronic survey to RTs within their department. The Canadian RT department manager list was acquired through the CAMRT and through network connections within the RT community. The Norwegian RT department managers were contacted directly. The survey was open for four weeks. Two weeks prior to closing the survey, the RT department managers were asked to distribute reminder emails to potential respondents. Data collection was completed in mid‐February 2021.

### Survey development

A separate cross‐sectional survey for RTs and RT department managers was developed. The development of the surveys was evidence‐informed^9^, ^10^, ^11^ and used feedback received from RT faculty members. Given the busy workload of RTs and limited time to complete surveys, a focused survey was developed to assess the impact of COVID‐19 specifically in the context of RT practice rather than using existing multi‐item tools to measure each construct of interest. The surveys were first created in English and then translated to French for the French‐speaking Canadian RTs and to Norwegian for the Norwegian RTs. A bilingual speaker (Norwegian and English) reviewed the translated surveys for accuracy, while the French translation service included back‐translation to English to verify the accuracy of the survey items. Prior to distribution, the survey was piloted by faculty members of the RT programme at the University of Alberta and Oslo Metropolitan University (OsloMet), selected RTs from Alberta, Ontario and British Columbia and by one RT department manager at the RT department of the Oslo university hospital. The pilot survey explored face validity which included clarity, ease of completion and coverage of key content domains. Changes were made based on feedback received. For the Canadian survey, data were collected anonymously using REDCap (version 10.6.2), a secure, web‐based application hosted by the Women and Children’s Health Research Institute at the University of Alberta. For the Norwegian survey, data were collected anonymously using Nettskjema (version 786, University of Oslo, Norway).

#### Survey for radiation therapists

The RT survey included 26 closed and five open‐ended questions and took approximately 10 to 13 minutes to complete. The closed questions asked participants to indicate their degree of agreement or disagreement with each survey item using a five‐point Likert scale. The open‐ended questions allowed participants to input text to express unaddressed concerns; thus, providing further insight into the psychological impact of the pandemic on RTs. The survey contained four categories: (1) demographics, (2) work‐related stressors due to COVID‐19, (3) work‐related psychological impact (stress, anxiety, depression, and insomnia) and impact on quality of life (QOL) due to COVID‐19, and (4) the availability and use of support programmes in the workplace (Table 1).

**Table 1:** Questions from survey administered to RTs in Canada and Norway.

**Question category**	**Questions**
Demographics	Are you a Canadian/Norwegian radiation therapist currently working in a Canadian/Norwegian radiation therapy department? a. If yes, were you working as a radiation therapist before October 1, 2019? Are you a radiation therapy manager? a. If yes, link to the survey for RT managers (Table 2) What is your radiation therapy role? What is your age? What is your gender? What province/county do you work in? Since March 2020, have you been working full‐time or part‐time? What are your years of working experience in radiation therapy? What is your level of education?
Work‐related stressors due to COVID‐19	Which of the following factors, if any, are impacting you psychologically? Changes in staffing due to COVID‐19 Changes in patient volumes due to COVID‐19 Changes in PPE usage due to COVID‐19 Changes in household income due to COVID‐19 Fear of transmitting COVID‐19 into workplace Fear of transmitting COVID‐19 from work to family and friends Fear of acquiring COVID‐19 from work
Work‐related psychological impact due to COVID‐19	I have experienced work‐related stress due to COVID‐19 (on a scale of 1–5). I have experienced work‐related anxiety due to COVID‐19 (on a scale of 1–5). I have experienced work‐related depression due to COVID‐19 (on a scale of 1–5). I have experienced work‐related insomnia due to COVID‐19 (on a scale of 1–5). Are there any other mental health issues that you are experiencing due to working in a COVID‐19 environment? Do you feel that the psychological impact of the COVID‐19 pandemic has affected your quality of life?
Availability and use of support programmes at the workplace	In my workplace, there are support programmes for dealing with psychological issues due to COVID‐19. I have accessed workplace support programmes for dealing with psychological issues due to COVID‐19. Are there any other forms of support you are using to deal with psychological issues due to COVID‐19?

#### Survey for RT department managers

The survey consisted of 42 closed and 13 open‐ended questions and took approximately 15 to 18 minutes to complete. The questions contained four categories: (1) departmental changes, (2) patient volume, (3) staff shortages and redeployment, and (4) personal protective equipment (PPE) and infection control measures (Table 2). In addition, the participants were able to share any additional thoughts or feedback.

**Table 2:** Questions from survey administered to RT department managers in Canada and Norway.

**Question category**	**Questions**
Departmental changes	Please indicate which measures were implemented for patients on treatment to reduce the risk of the COVID‐19 pandemic. Please indicate which treatment related or departmental policy changes were implemented due to the COVID‐19 pandemic.
Patient volume	How did the patient volume in your department change during the first wave (March to May 2020) of the COVID‐19 pandemic? How did the patient volume in your department change during the second wave (since October 2020 to present) of the COVID‐19 pandemic?
Staff shortages and redeployment	Were there any staff shortages in your department during the first wave (March‐May 2020) of the COVID‐19 pandemic? If yes, please specify the reasons for the staff shortages. If yes, how has the shortage of staff been addressed? (Please specify) Were there any staff shortages in your department during the second wave (October* 2020) of the COVID‐19 pandemic? If yes, please specify the reasons for the staff shortages. If yes, how has the shortage of staff been addressed? (Please specify) Did you have staff redeployed during the COVID‐19 pandemic?
PPE and infection control measures	Has training in the correct use of PPE in relation to COVID‐19 and infection control training been provided to all staff? (PPE = e.g., medical masks, eye protection, disposable gowns, etc.) Are there clear PPE guidelines established in your department? Do you feel that your employees have been affected by the infection control measures that have been implemented in your department due to the COVID‐19 pandemic?

### Ethics

Ethics approval was obtained from the Health Research Ethics Board of the Alberta Cancer Committee (HREBA‐CC for the Canadian portion of the study. As the collected data did not consist of information that could identify the participants, an approval from The Regional Committee for Medical and Health Research Ethics (REK) was not necessary for the Norwegian survey in accordance with the Norwegian Centre for Research Data. All responses were confidential and anonymous with no identifiable data elements. Informed consent was implied by completion of the survey by study participants.

### Data analysis

Descriptive analysis on all study variables was conducted to provide details on the study sample. Distributions of percentages were calculated for categorical variables and means and standard deviations for Likert scale survey items. Differences in the sample distributions of categorical variables between Norwegian and Canadian RTs were examined using chi‐square tests, and differences in the means of Likert scale survey items were examined using independent sample t‐tests. Logistic regression models examined the association between demographics (age, gender, work hours, work experience, education, work position) and presence of work‐related stressors due to COVID‐19 and the probability of reporting negative impact on QOL (outcome). Models were run separately for Norwegian and Canadian data. Fully adjusted odds ratios and 95% confidence intervals were calculated. Analyses were conducted using SPSS version 27. A p‐value <0.05 was considered significant throughout the study. The open‐ended questions were examined through thematic analysis according to the Braun and Clarke (2013) process,^15^ whereby after an initial review of the comments, the qualitative data were assigned codes that were then categorised into relevant themes. Qualitative comments from Norwegian RTs were translated to English for analysis and publication. A similar volume of responses was obtained from Norwegian and Canadian RTs, and the representative quotations selected to illustrate the final themes were drawn from both samples. Due to the specific content of the small number of respondents to the RT manager survey, it was not feasible to perform thematic analysis and maintain anonymity.

## Results

### RT survey

#### Demographics

This survey recorded 124 (approximate 41% response rate) and 155 (approximate 8% response rate) valid responses from Norway and Canada, respectively, with responses spanning nine counties in Norway and across six provinces in Canada (Table 3). Reasons for non‐participation among both RTs and managers from the viewpoint of department managers was a lack of sufficient time and suitable place with access to complete the survey. Ninety‐five percent of Norwegian respondents had a bachelor’s degree with one‐year specialisation in radiation therapy compared to 77% of Canadian respondents with a bachelor’s degree (Table 4). Most Norway RTs identified as being in a pre‐treatment position and dosimetrists (41%), while most Canadian RTs identified as treatment technologists (68%).

**Table 3:** Location of Norwegian and Canadian RT survey respondents.

**Location**	**%**
**Norwegian Counties (n = 124)**	
Agder	6
Innlandet & Trondelag	6
Møre og Romsdal	8
Nordland & Troms og Finnmark	9
Oslo	48
Rogaland	6
Vestland	17
**Canadian Provinces (*n* = 155)**	
Alberta	27
British Columbia	21
Nova Scotia & New Brunswick	11
Ontario	36
Quebec	4

Some counties and provinces were merged due to the small number of respondents from these locations.

**Table 4:** Demographic characteristics, presence of work‐related stressors due to COVID‐19, and psychological impact due to COVID‐19 reported by Norwegian and Canadian RTs.

**Study variables**	**Norway (*n* = 124)**	**Canada (*n* = 155)**
	**%**	**%**
**Age**		
<30 years	12	22
30–39 years	32	39
40–49 years	28	23
50+ years	25	17
Prefer not to say	2	–
**Gender**		
Male	19	15
Female	79	83
Prefer not to say	2	3
**Hours**		
Full‐time	82	80
Part‐time	18	20
**Years of working experience**		
0–5	15	27
6–10	22	19
11–15	16	18
16–20	20	12
21–25	13	15
25+	14	9
Education*		
Diploma	–	10
Bachelor’s degree	95	77
Master’s degree or higher	5	12
Prefer not to say	–	1
Position*		
Pre‐treatment and dosimetry	41	13
Treatment therapist	39	68
Educator	11	5
Other	9	15
**Presence of work‐related stressors due to COVID‐19**		
Changes in staffing due to COVID‐19	40	41
Changes in patient volumes due to COVID‐19	13	21
Changes in PPE usage due to COVID‐19	29	59*
Changes in household income due to COVID‐19	–^a^	20
Fear of transmitting COVID‐19 into workplace	68	52*
Fear of transmitting COVID‐19 from work to family/friends	57	76*
Acquiring COVID‐19 from work	34	59*

**Psychological impact due to COVID‐19 (scale 1–5)**	**Mean (SD)**	**Mean (SD)**
Work‐related stress due to COVID‐19	3.0 (1.3)	4.2 (0.8)*
Work‐related anxiety due to COVID‐19	2.2 (1.1)	3.9 (1.1)*
Work‐related insomnia due to COVID‐19	1.9 (1.0)	2.8 (1.3)*
Work‐related depression due to COVID‐19	2.0 (1.0)	2.8 (1.2)*

Chi‐square significant differences for categorical variables and independent sample t‐test for significant mean differences between Canadian and Norwegian respondents, *P < 0.05.

a Value is suppressed due to small cell count.

#### Work‐related stressors and psychological impact due to COVID‐19

The main work‐related stressor from COVID‐19 that impacted the psychological well‐being of RTs was fear of COVID‐19 transmission (Table 4). A higher percentage of Norwegian RTs had a fear of transmitting COVID‐19 into the workplace (68%) compared to Canadian RTs (52%), while a higher percentage of Canadian RTs had a fear of transmitting COVID‐19 from work to family and friends (76%) compared to Norwegian RTs (57%). Canadian RTs were also more likely to state that there were changes in PPE usage (59%) and have a fear of acquiring COVID‐19 from work (59%) compared to Norwegian RTs (29% and 34%, respectively).

Both Canadian and Norwegian RTs reported work‐related stress due to COVID‐19 as having the highest psychological impact from the pandemic (mean = 4.2; SD = 0.8 and mean = 3.0; SD = 1.3, respectively, on a five‐point scale) (Table 4). Canadian RTs reported significantly higher mean ratings for all psychological impacts of COVID‐19 compared to Norwegian RTs. Three themes were identified from the qualitative feedback related to the impact of working during the pandemic: generalised anxiety, such as due to fear of acquiring or transmitting COVID‐19; physical, emotional and cognitive symptoms of stress, such as mental exhaustion, fatigue and frustration; and loneliness, such as due to limited interactions with coworkers and family/friends due to fear of transmission (Table 5).

**Table 5:** Themes and representative quotations from the free‐text answers given by Norwegian and Canadian RTs.

**Question category**	**Theme**	**Representative quotations**
*Describe mental health issues that you are experiencing due to working during COVID‐19 pandemic*.	1. Generalised anxiety	*Generally worried about bringing it [COVID‐19] to loved ones as an asymptomatic carrier. Fear of passing it to a patient if unknowingly affected*. *I have anxiety going to work, I never get time alone, there is nowhere to eat or read alone*. *It [COVID‐19] has consumed my consciousness*.
	2. Physical, emotional, & cognitive symptoms of stress	*Stress from coworkers who are extremely anxious about COVID‐19*. *Suffering from impaired attention span; mental exhaustion*. *Trying to keep a vulnerable group (cancer patients) safe is causing stress*.
	3. Loneliness	*Feel isolated, and worried to go out and pass virus to someone*. *Feelings of isolation due to others' perception of health care workers being ‘high risk,’ i.e., friends not wanting to see me because of my high patient load*. *Feel like just not interacting with outside, staying home, doing nothing*.
*Describe how COVID‐19 has affected your quality of life*.	1. Negatively impacted physical and mental health	*I was essentially exhausted at the end of the day*. *The stress felt from the pandemic has affected my quality of sleep—harder to fall asleep, still feeling fatigued when I wake up*. *Not directly the impact on myself, but the psychological impact it has had on the people around me has affected my quality of life*.
	2. Quality of relationships	*I miss seeing my patients’ faces, and my coworkers faces ‐ seeing their emotions is such an important part of my job*. *Loss of work‐life balance and stress meant that my family did not get the best of me*. *Missing social outlets with coworkers. My work friendships have changed as we don't get to bond over out‐of‐work activities*.

#### Negative impact on QOL due to COVID‐19

When respondents were asked whether the psychological impact of COVID‐19, including stress, anxiety, depression, insomnia, had affected their quality of life, 80% of Canadians RTs and 47% of Norwegian RTs reported negative impact from the pandemic. The association between demographics and the presence of work‐related stressors on the probability of reporting negative impact on QOL due to COVID‐19 is shown in Table 6. Among Norwegian RTs, changes in PPE usage due to COVID‐19 were associated with a negative impact on QOL (OR = 4.17) in a model adjusting for all demographics and other stressors. Those who reported changes in PPE usage were over four times more likely to report negative impact on their QOL than those who did not experience changes in PPE usage. Among Canadian RTs, changes in staffing due to COVID‐19 were associated with a negative impact on QOL (OR = 3.23). Those who reported changes in staffing were over three times more likely to report negative impact on their QOL than those who did not report changes in staffing. As well, female Canadian RTs were significantly more likely to report that the pandemic negatively impacted their QOL (OR = 4.90) compared to male Canadian RTs, while Canadian RTs with 6–10 years of work experience were less likely to report negative impact on their QOL (OR = 0.12) compared to those with less than six years of work experience. Location of practice was not significantly associated with reporting negative impact on the QOL due to COVID‐19 (analysis not shown, estimates are available upon request). Two themes were identified from the qualitative feedback related to how COVID‐19 has affected QOL: negative impact on physical and mental health, such as causing poor quality of sleep and exhaustion from work; and negative impact on the quality of relationships, such as due to work‐life balance affecting family life and lack of opportunities to cultivate work friendships (Table 5).

**Table 6:** Adjusted odds ratios (OR) and 95% confidence intervals (CI) for the probability of reporting negative impact on quality of life (QOL) by demographics and presence of work‐related stressor due to COVID‐19 among Norwegian and Canadian RTs.

	**Norway**		**Canada**	
	**QOL**		**QOL**	
	**OR**	**95% CI**	**OR**	**95% CI**
**Age**				
<30 years	0.19	(0.01–3.19)	0.13	(0.01–4.27)
30–39 years	1.53	(0.24–9.87)	0.61	(0.03–12.48)
40–49 years	2.60	(0.48–14.23)	0.27	(0.03–2.94)
50+ years	*ref*		*ref*	
**Gender**				
Male	*ref*		*ref*	
Female	0.45	(0.13–1.55)	**4.90**	**(1.24–19.38)**
**Hours**				
Full‐time	*ref*		*ref*	
Part‐time	1.13	(0.34–3.81)	0.55	(0.14–2.12)
**Years of working experience**				
0–5	*ref*		*ref*	
6–10	0.84	(0.11–6.42)	**0.12**	**(0.02–0.93)**
11–15	0.70	(0.08–6.39)	0.58	(0.05–6.76)
16–20	0.65	(0.05–8.60)	0.55	(0.03–9.12)
21–25	0.53	(0.04–7.29)	0.96	(0.05–18.83)
25+	1.51	(0.10–24.17)	0.25	(0.01–9.29)
**Education**				
Diploma	–	–	0.21	(0.01–4.96)
Bachelor’s degree	*ref*		0.14	(0.01–2.04)
Master’s degree or higher	0.17	(0.01–2.70)	*ref*	
**Position**				
Pre‐treatment and Dosimetry	0.79	(0.25–2.50)	1.31	(0.22–7.92)
Treatment Therapist	*ref*		*ref*	
Educator	1.20	(0.23–6.18)	0.12	(0.01–1.65)
Other	0.71	(0.14–3.70)	3.04	(0.54–17.20)
**Presence of work‐related stressors due to COVID‐19**				
Changes in staffing due to COVID‐19	2.57	(0.93–7.07)	**3.23**	**(1.05–9.92)**
Changes in patient volumes due to COVID‐19	1.30	(0.29–5.77)	2.94	(0.59–14.69)
Changes in PPE usage due to COVID‐19	**4.17**	**(1.33–13.09)**	0.76	(0.28–2.06)
Transmitting COVID‐19 into workplace	1.53	(0.46–5.05)	1.24	(0.38–4.04)
Transmitting COVID‐19 from work to family/friends	0.85	(0.24–3.04)	2.06	(0.53–8.11)
Acquiring COVID‐19 from work	0.88	(0.27–2.84)	1.12	(0.34–3.68)

Bolding indicates significant associations, *P* < 0.05.

The reference category used for each association is provided in the table using *‘ref’*.

Models are fully adjusted for all other variables in table. Reported changes in household income are not included due to small cell counts.

#### Availability and use of support programmes in the workplace

Most Canadian RTs reported that support programmes for dealing with psychological issues due to COVID‐19 were available at their workplace (60%) compared to only 8% of Norwegian RTs. Although, 69% of Norwegian RTs were unsure if support programmes were available versus 33% of Canadian RTs. Most respondents indicated that they have not accessed support programmes (56% in Canada and 77% in Norway). From qualitative feedback, strategies for support outside the workplace for Norwegian RTs included staying connected with their social network, family, friends, and pets, as well as seeking professional help, including therapy and counselling. Canadian RTs mainly named recreational activities, such as exercise, hobbies, and meditation/ mindfulness, as well as some naming food, marijuana, and alcohol as forms of coping.

### Survey for RT managers

#### Departmental changes

Norwegian (*n* = 13) and Canadian (*n* = 12) RT department managers reported that they implemented visitor restrictions, physical distancing, screening and COVID‐19 testing of patients as measures for infection control.

#### Patient volumes

Some reported a decrease in patient volumes was reported in the first wave of the pandemic (March to May 2020) both in Norway (6/13, 46% of respondents) and Canada (8/12, 67% of respondents). In the second wave (October 2020 to January 2021) patient volumes were reported as unchanged by the majority of respondents in Norway (10/13, 77%), and Canada (7/12, 58%).

#### Staff shortages and redeployment

Staff shortages reported in the first wave were 39% (5/13) in Norway and 25% (3/12) in Canada and 31% (4/13) in Norway and 17% (2/12) in Canada in the second wave of the pandemic. Redeployment of staff during the pandemic was reported by a small number of respondents in both countries: 15% (2/13) in Norway and 33% (4/12) in Canada.

#### PPE and infection control measure

All respondents reported that RTs in their department were given training in the correct use of PPE, and those clear PPE guidelines were established. Approximately one‐third of managers in both countries reported that their employees have negatively been affected by the infection control measures that have been implemented in their department due to the COVID‐19 pandemic (5/13, 39% in Norway and 4/12, 33% in Canada).

## Discussion

### Key implications from RT surveys

The Norwegian and Canadian RT samples were similar in demographics with some exceptions related to a higher number of master’s degree holders and more specialised working positions for the Canadian RT group as compared to Norway. The results showed that Canadian RTs in the current study reported higher levels of work‐related stress, anxiety, depression, and insomnia than the Norwegian RTs. Fear of transmission, changes in PPE usage and changes in staffing due to COVID‐19 were reported as the most frequent work‐related stressors among RTs. Changes in PPE usage were most important to the QOL of Norwegian RTs, while changes in staffing were most important to the QOL of Canadian RTs.

Worrying about infecting friends and family members was reported by HCPs during the SARS epidemic.^16^, ^17^ A systematic review conducted in 2020 found that fear of transmission among HCPs was a source of increased anxieties.^18^ Fear of being infected has also been reported as a major concern by HCPs during the COVID‐19 pandemic.^9^, ^10^, ^19^, ^20^ These findings correspond with the current study findings that RTs are being impacted psychologically by the fear of transmitting COVID‐19 into their workplace, the fear of transmitting COVID‐19 from their workplace to friends and families, as well as the fear of acquiring COVID‐19. Of further interest would be an examination of the longitudinal changes in fear that may have ensued as the current understanding of the aetiology of the virus improves.

A recent scoping review also identified PPE as a stressor during the pandemic, particularly the extreme shortages in PPE supply, as well as the physical impact of continual use, such as dermatological symptoms and headaches.^21^ Similarities can be seen in this current study findings of Canadian and Norwegian RTs being affected psychologically by changes in PPE usage. Furthermore, healthcare systems across the world were faced with staff shortages during the pandemic.^21^, ^22^ While healthcare organisations have implemented creative strategies, such as extending the role of health professional students, staffing pressures remain high in many jurisdictions.^21^, ^22^, ^23^ The current study similarly found that staffing changes due to COVID‐19 was a commonly reported work‐related stressor among all RTs and associated with negative impact on QOL among Canadian RTs. As well, the increased prevalence of COVID‐19 in Canada as compared to Norway over this time may have attributed to finding higher levels of work‐related stress, anxiety, depression, and insomnia among Canadian RTs than the Norwegian RTs.

Studies from the SARS epidemic demonstrated that the effects of social isolation were the most potent in terms of provoking stress among HCPs.^24^, ^25^, ^26^ In the current study, loneliness emerged as a resounding theme from the qualitative feedback in terms of affecting the mental health of RTs, as well as a theme that represented the negative impact of COVID‐19 on the quality of existing relationships that affected the QOL of RTs.

The psychological impact of practicing during the SARS outbreak included elevated levels of stress, anxiety, and depression amongst HCPs.^1^, ^3^ Similarly, the COVID‐19 pandemic has been found to increase the levels of anxiety, depression, and insomnia amongst HCPs.^5^, ^6^ and increase levels of anxiety and stress amongst RTs.^9^, ^10^, ^11^ It has also been found that women have an increased risk of experiencing psychological symptoms.^6^, ^8^ The current study found that work‐related stress and anxiety were prevalent and that women and those with less than six years of work experience were more likely to have experienced a negative impact on their QOL. These findings are particularly important, given that previous studies have shown that the strain of practicing during a pandemic not only can lead to the development of mental health symptoms but also the worsening of pre‐existing psychiatric conditions.^27^, ^28^, ^29^ Post‐traumatic stress symptoms (PTSS) have also been reported in HCPs during a pandemic and may be exacerbated over longer periods of time.^7^

Published evidence demonstrates the importance of adequate mental health support programmes in the workplace to mitigate the psychological impact of the COVID‐19 pandemic on HCPs.^9^, ^10^, ^11^, ^30^ Disaster mental health interventions can include formal psychiatric treatment, and wellness‐ and resilience‐based psychosocial interventions.^31^, ^32^ Knowledge and utilisation of the support programmes offered by the workplace were minimal among both Norwegian and Canadian RTs. It is imperative to not only develop sustainable and accessible psychosocial support services in the workplace but also to ensure widespread knowledge of these resources among all HCPs within healthcare organisations.

### Key implications from RT department managers

The findings from the RT department managers survey suggest that the COVID‐19 pandemic had some organisational impact on the RT departments in both Norway and Canada due to implemented infection control measures and changes in staffing. Similar results have been found among British and Australian RTs and radiographers, where the pandemic has presented changes in the work environment, such as more uncertainty at work and major changes to routine departmental protocols.^9^, ^11^ Changes in work practices have likely contributed to a perceived general increase in work‐related stress.^9^ It is likely that a changed work environment has contributed to the work‐related stress among Canadian and Norwegian RTs as well.

### Limitations and future research

While the current study assessed several work‐related stressors from the pandemic, there was a limited description of each stressor and further nuances related to each stressor were lacking. As a result, there may have been differences in the possible interpretation of some stressors. For example, when assessing the impact of change in PPE usage, respondents may have interpreted this question as inquiring about the increased requirement for PPE, or change in the type of required PPE, or related to the concern over the amount of PPE available. Similarly, the question regarding the impact of change in staffing may have been interpreted as the number of staff available, or changes in staff roles due to redeployment. Therefore, further research could include qualitative in‐depth interviews or focus groups to capture a detailed narrative of the experience of RTs practicing during the pandemic. Although there was a similar number of respondents from both countries, the Canadian response rate was low accounting for approximately 8% of practicing RTs and the respondents did not represent all provinces. Also, the calculated response rate is an estimate since the number of practicing RTs at one time is difficult to determine because registration with a national body is not required to practice. In addition, a longitudinal study is needed to examine any changes in psychosocial impact as knowledge about the aetiology of the virus improved, as well as with increased access to vaccines. Further research that includes an exploration of the societal and cultural contexts is also needed to understand why the pandemic appears to have had a greater psychological impact on the Canadian RTs as compared to the Norwegian RTs.

## Conclusion

The current study suggests that the COVID‐19 pandemic has led to similar stressors amongst Canadian and Norwegian RTs but relatively higher levels of work‐related stress, anxiety, depression, and insomnia among Canadian RTs than Norwegian RTs. Fear of transmission, changes in PPE usage, and changes in staffing were reported as the most frequent work‐related stressors among RTs. Regardless of geography, workplace psychosocial supports must be available to HCPs to ensure minimised impact of these identified stressors.

## Conflict of Interest

The authors declare no conflict of interests.

## References

[1] Nickell LA , Crighton EJ , Tracy CS , et al. Psychosocial effects of SARS on hospital staff: survey of a large tertiary care institution. CMAJ 2004; 170: 793–8. 14993174 10.1503/cmaj.1031077 PMC343853

[2] Bai Y , Lin C‐C , Lin C‐Y , Chen J‐Y , Chue C‐M , Chou P . Survey of stress reactions among health care workers involved with the SARS outbreak. Psychiat Serv 2004; 55: 1055–7. 10.1176/appi.ps.55.9.1055 15345768

[3] Lee AM , Wong JG , McAlonan GM , et al. Stress and psychological distress among SARS survivors 1 year after the outbreak. Can J Psychiat 2007; 52: 233–40. 10.1177/070674370705200405 17500304

[4] Liu X , Kakade M , Fuller CJ , et al. Depression after exposure to stressful events: lessons learned from the severe acute respiratory syndrome epidemic. Compr Psychiat 2012; 53: 15–23. 21489421 10.1016/j.comppsych.2011.02.003 PMC3176950

[5] Pappa S , Ntella V , Giannakas T , Giannakoulis VG , Papoutsi E , Katsaounou P . Prevalence of depression, anxiety, and insomnia among healthcare workers during the COVID‐19 pandemic: a systematic review and meta‐analysis. Brain Behav Immun 2020; 88: 901–7. 32437915 10.1016/j.bbi.2020.05.026 PMC7206431

[6] Huang L , Wang Y , Liu J , et al. Factors influencing anxiety of health care workers in the radiology department with high exposure risk to COVID‐19. Med Sci Monitor 2020; 26: e926008. 10.12659/MSM.926008 PMC7401832 32710536

[7] Carmassi C , Foghi C , Dell'Oste V , et al. PTSD symptoms in healthcare workers facing the three coronavirus outbreaks: what can we expect after the COVID‐19 pandemic. Psychiatry Res 2020; 292: 113312. 32717711 10.1016/j.psychres.2020.113312 PMC7370915

[8] Lai J , Ma S , Wang Y , et al. Factors associated with mental health outcomes among health care workers exposed to coronavirus disease 2019. JAMA Netw Open 2020; 3: e203976. 32202646 10.1001/jamanetworkopen.2020.3976 PMC7090843

[9] Akudjedu TN , Lawal O , Sharma M , et al. Impact of the COVID‐19 pandemic on radiography practice: findings from a UK radiography workforce survey. BJR| Open 2020; 2: 1. 10.1259/bjro.20200023 PMC7583354 33178980

[10] Elshami W , Akudjedu TN , Abuzaid M , et al. The radiology workforce's response to the COVID‐19 pandemic in the Middle East, North Africa and India. Radiography (Lond) 2021; 27: 360–8. 33032889 10.1016/j.radi.2020.09.016 PMC7510634

[11] Shanahan MC , Akudjedu TN . Australian radiographers' and radiation therapists' experiences during the COVID‐19 pandemic. J Med Radiat Sci 2021; 68: 111–20. 33590670 10.1002/jmrs.462 PMC8013350

[12] Canadian Association of Medical Radiation Technologists (CAMRT) . COVID‐19 Health Human Resource Survey [Internet]. Canada: CAMRT; 21. October 2020 [downloaded 23. March 2021]. Available from: https://www.camrt.ca/wp‐content/uploads/2020/10/COVID‐19‐HHR‐Survey‐3‐%E2%80%93‐Summary‐Report.pdf

[13] Barua B , Hasan S , Comparing TI . Performance of Universal Health Care Countries, 2017. Fraser Institute.

[14] Georank . Canada vs Norway Coronavirus [Internet]. July 2021 [retrieved July 2021] from: https://georank.org/covid/canada/norway

[15] Braun V , Clarke V . Using thematic analysis in psychology. Qual Res Psychol 2006; 3: 77101.

[16] Elbay RY , Kurtulmuş A , Arpacıoğlu S , Karadere E . Depression, anxiety, stress levels of physicians and associated factors in Covid‐19 pandemics. Psychiatry Res 2020; 290: 113130. 32497969 10.1016/j.psychres.2020.113130 PMC7255248

[17] Cai H , Tu B , Ma J , et al. Psychological impact and coping strategies of frontline medical staff in Hunan between January and March 2020 during the outbreak of coronavirus disease 2019 (COVID‐19) in Hubei, China. Med Sci Monit 2020; 26: e924171. 32291383 10.12659/MSM.924171 PMC7177038

[18] Cabarkapa S , Nadjidai SE , Murgier J , Ng CH . The psychological impact of COVID‐19 and other viral epidemics on frontline healthcare workers and ways to address it: a rapid systematic review. Behavior, & Immunity –. Health 2020; 8: 100144. 10.1016/j.bbih.2020.100144 PMC7494453 32959031

[19] Li X , Yu H , Bian G , et al. Prevalence, risk factors, and clinical correlates of insomnia in volunteer and at home medical staff during the COVID‐19. Brain Behav Immun 2020; 87: 140–1. 32380272 10.1016/j.bbi.2020.05.008 PMC7198418

[20] Su TP , Lien TC , Yang CY , et al. Prevalence of psychiatric morbidity and psychological adaptation of the nurses in a structured SARS caring unit during outbreak: a prospective and periodic assessment study in Taiwan. J Psychiatr Res 2007; 41: 119–30. 16460760 10.1016/j.jpsychires.2005.12.006 PMC7094424

[21] Shaukat N , Mansoor Ali D , Ali RJ . Physical and mental health aspects of COVID‐19 on health care workers: a scoping review. International. J Emerg Med 2020; 13: 40. 10.1186/s12245-020-00299-5 PMC7370263 32689925

[22] Hester TB , Cartwright JD , DiGiovine DG , et al. Training and deployment of medical students as respiratory therapist extenders during COVID‐19. ATS Scholar Innov 2020; 1: 145–51. 10.34197/ats-scholar.2020-0049PS PMC8043304 33870278

[23] Rasmussen S , Sperling P , Poulsen MS , Emmersen J , Stig AS . Medical students for health‐care staff shortages during the COVID‐19 pandemic. The Lancet 2020; 395: e79–80. 10.1016/S0140-6736(20)30923-5 PMC7180031 32334649

[24] Banerjee D , Rai M . Social isolation in Covid‐19: the impact of loneliness. Int J Soc Psychiatry 2020; 66: 525–7. 32349580 10.1177/0020764020922269 PMC7405628

[25] Lu W , Wang H , Lin Y , Li L . Psychological status of medical workforce during the COVID‐19 pandemic: a cross‐sectional study. Psychiatry Res 2020; 288: 112936. 32276196 10.1016/j.psychres.2020.112936 PMC7195354

[26] Lin CY , Peng YC , Wu YH , Chang J , Chan CH , Yang DY . The psychological effect of severe acute respiratory syndrome on emergency department staff. Emerg Med J 2007; 24: 12–7. 17183035 10.1136/emj.2006.035089 PMC2658141

[27] McAlonan GM , Lee AM , Cheung V , et al. Immediate and sustained psychological impact of an emerging infectious disease outbreak on health care workers. Can J Psychiatry 2007; 52: 241–7. 17500305 10.1177/070674370705200406

[28] Hall RC , Hall RC , Chapman MJ . The 1995 Kikwit Ebola outbreak: lessons hospitals and physicians can apply to future viral epidemics. Gen Hosp Psychiatry 2008; 30: 446–52. 18774428 10.1016/j.genhosppsych.2008.05.003 PMC7132410

[29] Montemurro N . The emotional impact of COVID‐19: From medical staff to common people. Brain Behav Immun 2020; 87: 23–4. 32240766 10.1016/j.bbi.2020.03.032 PMC7138159

[30] Carmassi C , Cerveri G , Bui E , Gesi C , Dell’Osso L . Defining effective strategies to prevent posttraumaticstress in healthcare emergency workers facing the COVID‐19 pandemic in Italy. CNS Spectrums. 2020 Letter to the Editor 10.1017/S1092852920001637 PMC7642502 32660685

[31] North CS , Pfefferbaum B . Mental health response to community disasters: a systematic review. JAMA 2013; 310: 507. 23925621 10.1001/jama.2013.107799

[32] Shreffler J , Petrey J , Huecker M . The impact of COVID‐19 on healthcare worker wellness: a scoping review. Western J Emerg Med 2020; 21: 1059–66. 10.5811/westjem.2020.7.48684 PMC7514392 32970555

